# Editorial: Emerging Infectious and Vector-Borne Diseases: A Global Challenge, Volume II

**DOI:** 10.3389/fpubh.2022.942950

**Published:** 2022-05-31

**Authors:** Katherine M. Warpeha, Shu Hui Chen, Catherine Mullié

**Affiliations:** ^1^Department of Biological Sciences, University of Illinois at Chicago, Chicago, IL, United States; ^2^Office of Data Science Strategy, National Institutes of Health (NIH), Bethesda, MD, United States; ^3^Laboratoire AGIR (Agents Infectieux, Résistances et Chimiothérapie), EA UPJV 4294, Université de Picardie Jules Verne, Amiens, France

**Keywords:** infectious disease, vector-borne, zoonotic, viral, containment, preparedness, diagnostics, climate change

When the first volume of this Research Topic was published in early 2020, the COVID-19 pandemic was only just beginning, unfortunately increasing its contemporaneity. At the end of 2020, the Research Topic relaunched to include COVID-19 issues in its scope, along with challenges in identification, transfer, spread, treatment, and containment of other infectious and vector-borne (re-) emerging diseases. As a result, this second volume gathers five papers dealing with various present-day aspects involved in a global approach to tackle infectious diseases.

The impact of climate change on global health issues and, more specifically, on the seasonal and geographical spread of pathogens is still at the forefront of preoccupations in 2022, as underlined by the *Lancet* countdown, an international collaboration monitoring the consequences of climate change on health. These consequences are multiple and include the propagation of vectors such as mosquitoes, mites as well as sundry insects and arthropods. Researchers are experimenting with high-technology and sometimes controversial solutions such as genetically modified mosquitoes to control this propagation. Nevertheless, low technology alternatives facilitating public health preparedness should not be overlooked, especially in low income regions. For example, the article by Lu et al. investigated the role of meteorological factors during the rising incidence of scrub typhus (a bacterial disease transmitted by mites) witnessed in Southern China over the 2006–2018 period. They described non-linear and lagged relationships between the number of scrub typhus cases and factors such as the weekly mean temperature and relative humidity. The results provide epidemic trends, which could help in the development of early warning systems through meteorological factors surveillance and allow local health authorities to strengthen the prevention and control of this disease when meteorological threshold values are reached. On a larger scale, the review by Chala and Hamde explored the intricate reasons for (re-)emergence of vector-borne infectious diseases, especially zoonotic viral ones. The authors once more underline the direct impact of climate change on vector geographical distribution but also highlight its indirect influence through land use modifications. Such land use modifications can result from climatic factors but also from non-climatic ones, as indicated by the review. Hence, the authors underscore the need to take a common global stance to tackle the issue of vector dissemination and argue for an empowerment of the One Health approach.

Indeed, the control of vector dissemination is one of the major keys, if not the major key, to limit the spread of most emerging diseases. However, not all emerging diseases are vector-borne. In such cases, other preventative measures should be implemented, be it on a specific or a population scale. Examples of the efficiency of such preventative measures are provided in the studies by Cutts et al. and Benavides et al., both dealing with the transmission of Ebola virus. The first study demonstrated that wipes pre-impregnated with various disinfectants such as activated hydrogen peroxide, ethanol, sodium hypochlorite or a dual quaternary ammonium compound efficiently removed Ebola virus from stainless steel surfaces. These decontamination tools could therefore be useful to limit indirect transmission *via* high touch surfaces during outbreaks. The latter study took a population-based approach to limit the viral dissemination during the 2021 Ebola outbreak in Guinea and Liberia. Benavides et al. advocated for the use of music to raise public awareness and help in changing behaviors at both individual and population levels. Building on their practical experience, the authors propose a four-step template for the implementation and evaluation of a successful music-based health intervention. This kind of intervention could especially help in reaching young individuals within a given population and, if validated by health authorities, could be complementary to standard prevention messages issued by said health authorities.

Finally, the preparedness of major companies to face the global economic disruptions triggered by epidemics such as COVID-19 was dissected by MacDonald et al. Taking US 2018 Fortune 500 companies as an example, the authors point out that, pre-COVID-19, these non-traditional stakeholders in global health might have underestimated or even not recognized the significant impacts of infectious disease threats on their businesses. The apparent lack of awareness of media and motor companies is specifically pointed out. The authors conclude their analysis by advocating for an “all-of-society” approach to health security involving the private sector to better prevent, detect, and respond to economic disruptions related to infectious disease events.

We hope that the papers collected in this Research Topic will raise awareness on these scientific, demographic and socio-economic problems related to (re-)emerging infectious and vector-borne diseases, enabling the development of implementable practical solutions both at local and global scales.

## Author Contributions

All authors listed have made a substantial, direct, and intellectual contribution to the work and approved it for publication. KW, SC, and CM contributed as monitoring editors for this Research Topic.

## Author Disclaimer

The views expressed in this Editorial are those of the authors and do not represent the views of the U.S. National Institutes of Health; the U.S. Department of Health and Human Services; the University of Illinois at Chicago; or the Université de Picardie Jules Verne.

## Conflict of Interest

The authors declare that the research was conducted in the absence of any commercial or financial relationships that could be construed as a potential conflict of interest.

## Publisher's Note

All claims expressed in this article are solely those of the authors and do not necessarily represent those of their affiliated organizations, or those of the publisher, the editors and the reviewers. Any product that may be evaluated in this article, or claim that may be made by its manufacturer, is not guaranteed or endorsed by the publisher.

